# Clinical and genetic analyses in a patient with PAPA syndrome complicated with inflammatory bowel disease

**DOI:** 10.1186/1546-0096-13-S1-P148

**Published:** 2015-09-28

**Authors:** H Ida, Y Kunitake, N Yoshida, D Wakasugi, S Kaieda, K Mitsuyama, K Iwamoto, K Fujita, R Nishikomori

**Affiliations:** 1 Kurume University School of Medicine, Department of Medicine, Kurume, Fukuoka, Japan; 2 Kurume Hospital, Coloproctology Center, Kurume, Fukuoka, Japan; 3 Tenri Hospital, Department of Pathology, Tenri, Nara, Japan; 4 Kyoto University Graduate School of Medicine, Department of Pediatrics, Kyoto, Kyoto, Japan

## Introduction

PAPA (pyogenic arthritis, pyoderma gangrenosum and acne) syndrome is an autoinflammatory disease linked to mutations in the *PSTPIP1* gene. These mutations produce a hyper-phosphorylated PSTPIP1 protein and alter its participation in the activation of the “inflammasome”.

## Objectives

To elucidate the pathogenesis of PAPA syndrome, we examined the clinical status and complications and also analyzed the *PSTPIP1* gene.

## Patients and methods

We herein report a 23-year-old Japanese male who suffered from recurrent arthritis in his knee and ankle joints, pyoderma gangrenosum, and acne. Recently, he had experienced melena and multiple colonic ulcers had been detected by colonfiberscopy. His ulcerations resembled ulcers associated with Crohn's disease. A histological examination was then performed for the synovium of this knee joints, skin lesions of pyoderma gangrenosum, and the colon. The genomic DNA of *PSTPIP1* were analysed in both the patient and his family.

## Results

1) A histological analysis revealed that a large number of neutrophils had accumulated in the skin lesions; however, very few neutrophils were detected in the pathological lesions of the knee joints and colon. 2) According to a gene analysis, we detected a novel heterozygous mutation in the *PSTPIP1* gene; however, his healthy father also had the same mutation, thus suggesting that this mutation of *PSTPIP1* might not be related to his phenotype. We are searching for other affected genes besides the *PSTPIP1* gene for PAPA syndrome in this case.

## Conclusion

We herein reported a Japanese PAPA syndrome patient who was complicated with inflammatory bowel disease. A genetic analysis suggested that this particular phenotype might not have been affected by a mutation of the *PSTPIP1* gene.

